# Atorvastatin at Reperfusion Reduces Myocardial Infarct Size in Mice by Activating eNOS in Bone Marrow-Derived Cells

**DOI:** 10.1371/journal.pone.0114375

**Published:** 2014-12-03

**Authors:** Yikui Tian, Joel Linden, Brent A. French, Zequan Yang

**Affiliations:** 1 Department of Surgery, University of Virginia Health System, Charlottesville, Virginia, United States of America; 2 Department of Medicine, University of Virginia Health System, Charlottesville, Virginia, United States of America; 3 Department of Biomedical Engineering, University of Virginia Health System, Charlottesville, Virginia, United States of America; 4 La Jolla Institute for Allergy & Immunology, La Jolla, California, United States of America; 5 Department of Cardiovascular Surgery, Tianjin Medical University General Hospital, Tianjin, P.R. China; Thomas Jefferson University, United States of America

## Abstract

**Background:**

The current study was designed to test our hypothesis that atorvastatin could reduce infarct size in intact mice by activating eNOS, specifically the eNOS in bone marrow-derived cells. C57BL/6J mice (B6) and congenic eNOS knockout (KO) mice underwent 45 min LAD occlusion and 60 min reperfusion. Chimeric mice, created by bone marrow transplantation between B6 and eNOS KO mice, underwent 40 min LAD occlusion and 60 min reperfusion. Mice were treated either with vehicle or atorvastatin in 5% ethanol at a dose of 10 mg/kg IV 5 min before initiating reperfusion. Infarct size was evaluated by TTC and Phthalo blue staining.

**Results:**

Atorvastatin treatment reduced infarct size in B6 mice by 19% (p<0.05). In eNOS KO vehicle-control mice, infarct size was comparable to that of B6 vehicle-control mice (p = NS). Atorvastatin treatment had no effect on infarct size in eNOS KO mice (p = NS). In chimeras, atorvastatin significantly reduced infarct size in B6/B6 (donor/recipient) mice and B6/KO mice (p<0.05), but not in KO/KO mice or KO/B6 mice (p = NS).

**Conclusions:**

The results demonstrate that acute administration of atorvastatin significantly reduces myocardial ischemia/reperfusion injury in an eNOS-dependent manner, probably through the post-transcriptional activation of eNOS in bone marrow-derived cells.

## Introduction

Lipid-lowering therapy by 3-hydroxy-3-methylglutaryl-co-enzyme A (HMG-CoA) reductase inhibitors (i.e., statins), has largely been viewed as a long-term strategy to reduce cardiovascular risk. Recent studies suggested that early use of statins after acute coronary syndromes may reduce the risk of subsequent ischemic cardiovascular events, and the salutary effects of this early initiation of treatment was independent of baseline levels of cholesterol [1]–[3]. This suggests that, besides the lipid-lowering effects resulting from long-term use, statins might also act rapidly to reverse abnormalities of the circulatory system that may predispose to recurrent ischemic events. Potential examples of such abnormalities include endothelial dysfunction [4], [5], local inflammatory responses [6], [7], and/or an exaggerated thrombogenic tendency [8]. Several clinical trials have demonstrated that early statin treatment could reduce myocardial injury in patients undergoing PCI for myocardial infarction [9]–[11], although others reported opposite results [12]. However, the precise mechanisms of the infarct-sparing effect of statins remain to be defined. Animal studies have shown that statins, such as atorvastatin and simvastatin, attenuate myocardial I/R injury in a manner that is independent of lipid lowering effect [13], [14]. Furthermore, statin was recently found to exert cardioprotective effects when administered at the onset of reperfusion by activating a signal transduction pathway involving endothelial eNOS [15]. Recently, eNOS has been identified in human and mouse platelets [16], [17]. Statins, such as atorvastatin, increase eNOS levels in platelets in a dose-dependent manner and decrease platelet activation *in vivo* [16]. This inhibition of platelet activation through the upregulation of platelet eNOS may contribute to the atorvastatin-mediated protection against cerebral I/R injury [16]. The potential role of platelet eNOS in limiting myocardial I/R injury has yet to be explored.

In the current study, we examined the acute cardioprotection afforded by administering atorvastatin shortly before reperfusion in an intact mouse model of myocardial ischemia/reperfusion injury. We first hypothesized that atorvastatin acts as a potent inhibitor of post-ischemic inflammatory responses and thus protects the heart against reperfusion injury by activating eNOS. Given that the cardioprotective effects of atorvastatin proved to be robust in our model, we further examined the respective roles of endothelial eNOS and bone marrow-derived eNOS in atorvastatin-mediated cardioprotection.

## Materials and Methods

This study conformed to the Guide for the Care and Use of Laboratory Animals published by the National Institutes of Health (Eighth Edition, revised 2011) and was conducted under protocols approved by the University of Virginia's Institutional Animal Care and Use Committee (Protocol Number: 3985).

### Materials

Atorvastatin was a gift of Pfizer Inc. Atorvastatin was prepared in PBS and 5% (vol/vol) ethanol (pH 7.6). Triphenyltetrazolium chloride (TTC) was purchased from Sigma Chemical Co. (St. Louis, MO) and 3-diaminobenzidine tetrahydrochloride from DAKO, Inc. (Carpinteria, CA). Rat anti-mouse neutrophil antibody was purchased from Serotec, Inc. (Raleigh, NC) and rabbit polyclonal antibody against a peptide corresponding to the 25 COOH-terminal amino acids of P-selectin was a gift from Dr. S. A. Green (Univ. of Virginia, Charlottesville, VA).

### Animals

A total of 122 male, 9–14 week old mice, purchased from Jackson Laboratory (Bar Harbor, ME) were use in this study, including wild-type C57BL/6 (B6) mice and eNOS knockout (KO) mice. Among these, 71 mice were chimeras created between these two strains by bone marrow transplantation to post-irradiated recipients. These mice were assigned to 16 groups as detailed in Table 1.

**Table 1:** Animal groups and protocols.

Groups	Protocols		End-points	n	Mortality + Exclusion
Wild type (B6) & eNOS KO (KO)	B6 + Vehicle	I/R: 45′/60′	CBC	4	1 died
	B6 + Atorvastatin	I/R: 45′/60′	CBC	4	
	KO + Vehicle	I/R: 45′/60′	CBC	4	
	KO + Atorvastatin	I/R: 45′/60′	CBC	4	
	B6 + Vehicle	I/R: 45′/60′	IF	10	
	B6 + Atorvastatin	I/R: 45′/60′	IF	10	
	KO + Vehicle	I/R: 45′/60′	IF	7	
	KO + Atorvastatin	I/R: 45′/60′	IF	8	
Chimeras	B6/B6 + Vehicle	I/R: 40′/60′	IF&IHC	9	1 died and 1 exclusion
	B6/B6 + Atorvastatin	I/R: 40′/60′	IF&IHC	10	
	KO/B6 + Vehicle	I/R: 40′/60′	IF	9	
	KO/B6 + Atorvastatin	I/R: 40′/60′	IF&IHC	9	
	B6/KO + Vehicle	I/R: 40′/60′	IF	8	
	B6/KO + Atorvastatin	I/R: 40′/60′	IF&IHC	8	
	KO/KO + Vehicle	I/R: 40′/60′	IF	9	
	KO/KO + Atorvastatin	I/R: 40′/60′	IF	9	
Total	16 groups			122	2+1

I/R: ischemia/reperfusion, B6: C57BL/6, KO: eNOS knockout, CBC: complete blood count, IF: infarct size, IHC: immunohistochemistry. Atorvastatin was given intravenously 5 min before reperfusion at a dose of 10 mg/Kg.

### Bone marrow transplantation

Chimeras were produced using standard techniques as described previously [18], [19]. Briefly, donor mice (8∼10 wks; 24–26 g) were euthanized with an overdose of sodium pentobarbital. Death was then confirmed by cervical dislocation. The bone marrow from the tibia and femur was harvested under sterile conditions yielding ∼50 million nucleated bone marrow cells per mouse. Recipient mice (6∼7 wks; 22–25 g) were irradiated with two doses of 600 rads each, 4 hours apart. Immediately following irradiation, 2∼4×10^6^ bone marrow cells were injected intravenously via external jugular vein under general anesthesia plus local injection of Bupivacaine. During each irradiation, two control mice were included that did not receive bone marrow transplantation. Irradiated/transplanted mice were housed in micro-isolators for at least 8 weeks prior to experimentation.

### Hemodynamic studies in chimeras

Hemodynamic parameters were assessed in 3 mice from each group of chimeras. Mice were anesthetized with isoflurane (1% by volume in oxygen). The right common carotid artery was exposed and cannulated with a 1.4F Millar micro-tip catheter (Millar Instruments, Inc., Houston, TX). After acquiring peripheral arterial blood pressures, the catheter tip was advanced into left ventricular chamber. LV pressures (LVESP and LVEDP) and developed pressures (dP/dt + and -) were recorded (Table 2).

**Table 2:** Hemodynamics in chimeric mice.

Chimera	HR (bpm)	Arterial Pressure (mmHg)			LV Pressure (mmHg)		Developed Pressure (dP/dt, mmHg/s)	
(n = 3)		Sys	Dias	Mean	ESP	EDP	+dP/dt	- dP/dt
B6/B6	460±38	93±1	67±2	76±2	93±1	6±2	6611±223	6422±226
KO/B6	433±18	94±2	68±1	77±1	94±2	8±2	6377±255	6299±418
B6/KO	410±10	105±2*	72±1^†^	83±1*^†^	108±3*	7±1	7235±229	7167±185
KO/KO	417±9	112±3*	81±1*	91±1*	106±1*	6±2	6683±431	6768±345
*t* test	NS	**p<0.05**	**p<0.05**	**p<0.05**	**p<0.05**	NS	NS	NS

HR: heart rate, bpm: beats per min, Sys: systolic, Dias: diastolic, LV: left ventricle, ESP: end-systolic pressure, EDP: end-diastolic pressure. *p<0.05 vs. B6/B6 or KO/B6; ^†^p<0.05 vs. KO/KO.

### Myocardial ischemia/reperfusion

Mice were subjected to 40 or 45 min of coronary occlusion followed by 60 min of reperfusion and then euthanized to count peripheral white blood cells and to evaluate myocardial infarct size and/or leukocyte infiltration (Table 1). A standard protocol was employed, as detailed previously [19], [20]. Briefly, mice were anesthetized with sodium pentobarbital (80–100 mg/kg, IP) and orally intubated. Artificial respiration was maintained with a FiO2 of 0.80, 100 strokes/min, and a 0.3–0.5 ml stroke volume. The heart was exposed through a left thoracotomy and coronary artery occlusion was achieved by passing a suture beneath the LAD and tightening over a piece of PE-60 tubing for 40 or 45 min. Reperfusion was induced by removing the PE-60 tubing.

### Myocardial infarct size measurement

The mice were euthanized at 60 min after reperfusion and the hearts were cannulated through the ascending aorta for sequential perfusion with 3∼4 ml of 1.0% TTC and 10% Phthalo blue. The LAD was re-occluded with the same suture used for coronary occlusion prior to Phthalo blue perfusion to determine risk region. The LV was cut into 5–7 transverse slices that were weighed and digitally photographed for determining infarct size as a percent of risk region as described previously [19], [20].

### Peripheral blood cell counts (CBC)

Peripheral blood cells were counted in B6 and eNOS KO mice with or without atorvastatin treatment (4 each in each group). CBC was measured before LAD occlusion and again at 60 min post-reperfusion following the 45 min LAD occlusion. Blood (30–40 µl) was obtained by puncturing the left external jugular vein at each time point. Cell counts were performed with a HemaVet Hematology System (CDC Technologies, Oxford, CT).

### Immunohistochemistry of neutrophils and platelets

Hearts were harvested and cut into five to seven short-axis slices and immediately fixed in 4% paraformaldehyde in PBS (pH 7.4) for paraffin embedding. Paraffin-embedded sections (5 µm) were rehydrated and incubated with 1% hydrogen peroxide. After being rinsed in PBS, the sections were incubated with 10% blocking serum. Immunostaining was performed with the use of a rabbit polyclonal antibody (1∶4,000) against a peptide corresponding to the 25 COOH-terminal amino acids of P-selectin (a gift from Dr. S. A. Green at Univ. of Virginia, Charlottesville). A rat anti-mouse neutrophil antibody (1∶1,000) (Serotec, Raleigh, NC) was used to identify tissue neutrophils. The appropriate biotinylated secondary antibodies (Vector Laboratories) were then applied for 1 h at room temperature. After incubation with avidin-biotin complex (Vector Laboratories), immunoreactivity was visualized by incubating the sections with 3,3-diaminobenzidine tetrahydrochloride (DAKO) to produce a dark brown precipitate.

### Statistical analysis

All data are presented as the mean ± SEM. Cell counts, hemodynamic parameters, infarct sizes and risk region sizes were compared using one-way ANOVA followed by Student's t test with Bonferroni correction.

## Results

### Exclusion and mortality

Of the 122 mice that underwent myocardial ischemia/reperfusion injury, 2 mice died during early reperfusion. One mouse was excluded due to an inordinately small risk region (<25% of LV mass) (Table 1).

### Effects of atorvastatin on myocardial infarction in B6 and eNOS KO mice

**Figure 1:**
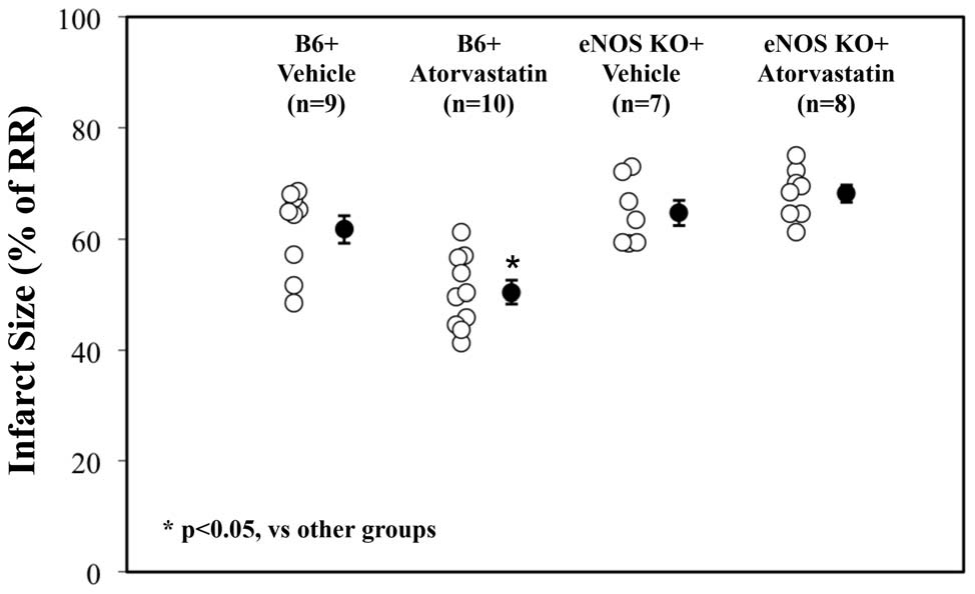
Myocardial infarct size in wild type and congenic eNOS KO mice. Myocardial infarct size (as percentage of risk region) was measured after 45 min of LAD occlusion and 60 min of reperfusion. Vehicle or atorvastatin was administered 5 min prior to the onset of reperfusion. Infarct size was significantly smaller in atorvastatin-treated B6 mice than in vehicle-treated B6 controls or either group of eNOS KO mice (p<0.05).

In WT C57BL/6 mice, 45 min of LAD occlusion and 60 min of reperfusion produced an infarct size of 62±2 (% of the risk region, RR). Atorvastatin administered 5 min before reperfusion resulted in a 19% decrease in myocardial infarct size as assessed by TTC staining (Fig. 1 & Data S1). A comparable infarct size was found in vehicle-treated eNOS KO mice (65±2, p = NS vs. B6 vehicle control,), however, atorvastatin provided no protection in eNOS KO mice (68±2 vs. 65±2, p = NS. See Fig. 1).

**Figure 2:**
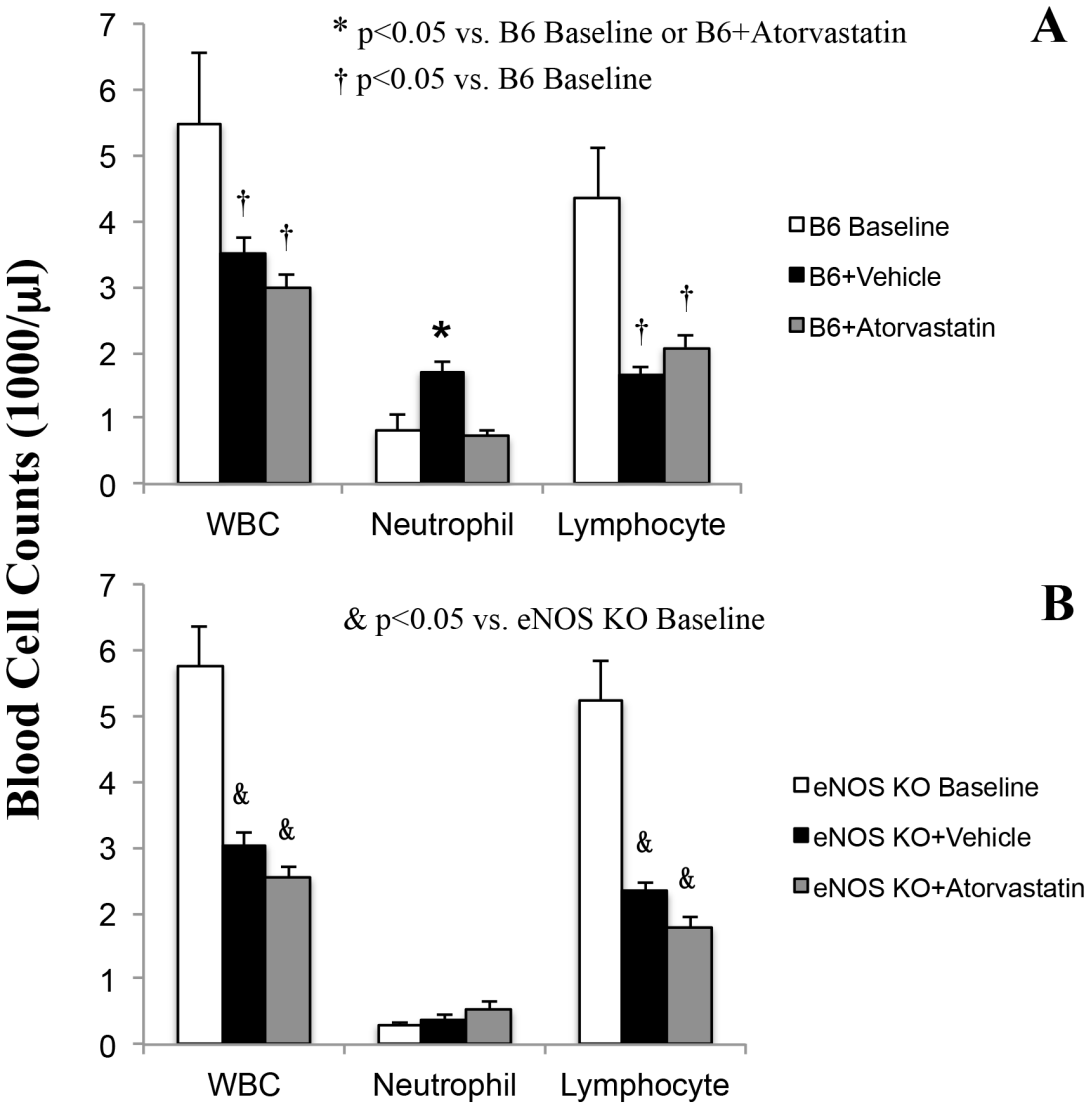
Blood cell differentials in B6 (Panel A) and eNOS KO (Panel B) mice. Blood samples were acquired before ischemia (open bar) and after 45 min of LAD occlusion and 60 min of reperfusion (black or shaded bars). At baseline, hemoglobin, WBC and platelets were comparable between B6 and eNOS KO mice; however, the white cell differentials showed significantly higher lymphocytes in eNOS KO mice. In B6 mice, LAD occlusion and reperfusion caused a 40–50% reduction in total circulating white blood cells and a 50–60% reduction in circulating lymphocytes. In contrast, the neutrophil count nearly doubled after ischemia/reperfusion in B6 mice, but this negative effect was essentially abolished atorvastatin when administered just prior to the onset of reperfusion.

In order to define the target(s) that atoravastatin acts upon to produce the cardioprotective effect, the effect of atorvastatin, as well as the eNOS gene, on blood cells were evaluated by HemaVet. Between B6 and eNOS KO mice, the hematology parameters were comparable in hemoglobin levels (12.9±0.2 vs. 12.5±0.2 g/dl), WBC count (5.5±1.1 vs 5.8±0.6 K/µl), and count of platelets (784±57 vs. 893±51 K/µl). However, the white blood cell differentials were significantly different between the two strains, neutrophils: 14% in B6 vs. 6% in eNOS KO mice and lymphocytes: 83% vs. 91% (p<0.05). In B6 mice, LAD occlusion and reperfusion caused a 40–50% reduction in total circulating white blood cells and a 50–60% reduction in circulating lymphocytes. In contrast, the neutrophil count nearly doubled after ischemia/reperfusion in B6 mice, but this negative effect was effectively abolished by atorvastatin when administered just prior to the onset of reperfusion (Fig 2 & Data S1).

### Hemodynamic parameters in chimeras

Mild hypertension has been found in eNOS KO mice [21]. Hemodynamic parameters in chimeric mice were assessed before studying these mice in the I/R injury protocol. Mice were anesthetized with 1% isoflurane. Arterial blood pressure and left ventricular pressure were measured with a Millar MicroTip Catheter in 4 different groups of chimeras (donor/recipient by definition). There were no significant differences in heart rate, left ventricular end-diastolic pressure or left ventricular developed pressures (Table 2). However, there was a 20% increase in peripheral arterial pressures (systolic, diastolic and mean pressures) and in left ventricular end-systolic pressure (LVESP) in KO/KO mice as compared to B6/B6 mice. In KO/B6 mice, where only circulating blood cells lacked eNOS activity, no changes were found in peripheral blood pressures and LVESP as compared to B6/B6 chimera. Whereas in B6/KO mice, where only circulating blood cells had eNOS activity, systolic and mean arterial pressures as well as LVESP remained significant higher than that of B6/B6 or KO/B6, but diastolic and mean arterial pressures were found to be significantly lower than that of KO/KO chimeras (Table 2).

### Infarct sparing effect of atorvastatin in chimeras

**Figure 3:**
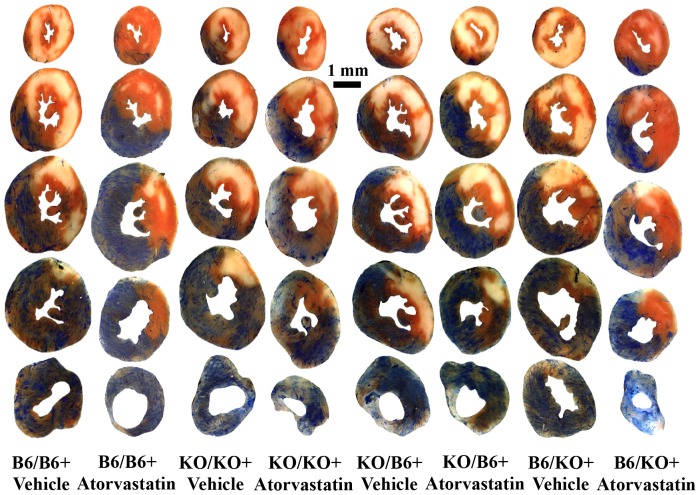
TTC- and Phthalo blue-stained, short-axis tissue sections of left ventricles from representative mice corresponding to the chimeric mice groups shown in Fig 3. Blue areas are non-ischemic tissue; yellowish-white areas are infarcted tissue; and red areas represent salvaged (viable) tissue within the ischemic regions.

**Figure 4:**
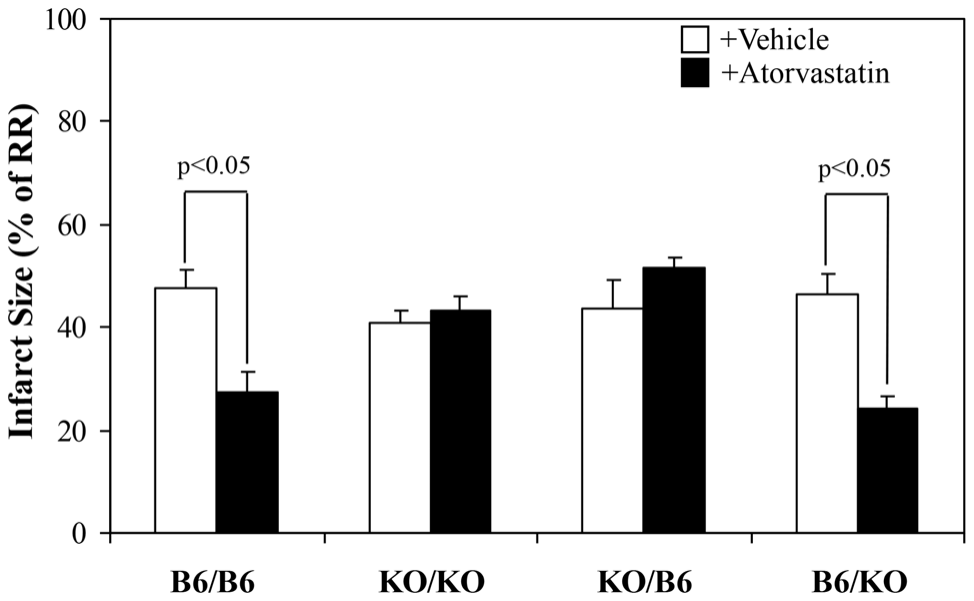
Myocardial infarct size in chimeric mice. Myocardial infarct size (as percent risk region) in chimeric mice was measured after 40 min of LAD occlusion and 60 min of reperfusion. Each chimeric mouse was treated either with vehicle or atorvastatin 5 min before reperfusion. As compared to the corresponding vehicle-treated control group, atorvastatin significantly reduced infarct size by 42% in B6/B6 mice and by 48% in B6/KO mice (p<0.05 for each vs. vehicle control). However, atorvastatin showed no cardioprotective effects in KO/KO or KO/B6 chimeras (p = NS).

**Figure 5:**
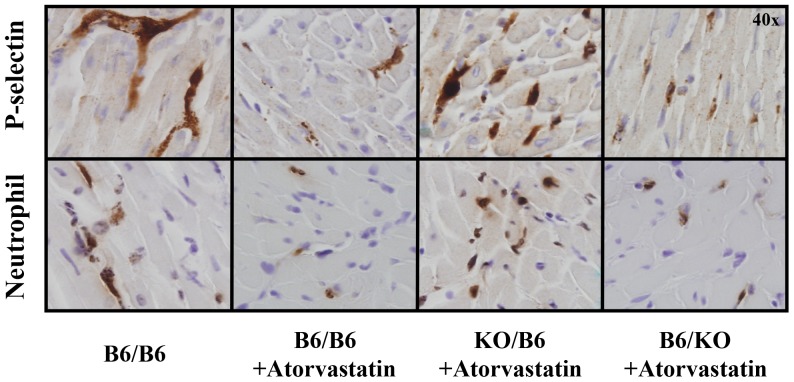
Effect of atorvastatin on platelet and neutrophil infiltration after reperfusion. The infiltration of platelets (upper panel) and neutrophils (lower panel) were evaluated by immunohistochemistry in the 4 chimeric groups that received 40 min of LAD occlusion and 60 min of reperfusion. Platelets and neutrophils were found predominantly in the ischemic area in B6/B6 chimeras. Atorvastatin reduced both platelet and neutrophil infiltration in B6/B6 and B6/KO chimeras, but not in KO/B6 chimeras.

As our ongoing studies revealed that the mouse model of ischemia/reperfusion injury can be made more sensitive to the effects of drug intervention on reperfusion injury by reducing the duration of ischemia from 45 min to 40 min, we refined our protocol accordingly by adopting 40 min of LAD occlusion and 60 min of reperfusion. Risk regions (RR, defined as percentage of left ventricular mass) were comparable among the 8 groups (35% to 43%, p = NS). Infarct size (% of RR) was also comparable among the vehicle-treated chimeras, which ranged from 41% to 48%. Compared to the corresponding vehicle-treated group, atorvastatin significantly reduced infarct size by 42% in B6/B6 mice and by 48% in B6/KO mice (p<0.05). However, no cardioprotective effect was found in KO/KO or KO/B6 chimeras (Figs 3, 4 & Data S1). Immunohistochemistry was performed in vehicle-treated B6/B6 mice, and atorvastatin-treated B6/B6, KO/B6 and B6/KO chimeras (n = 3 in each group) to evaluate the infiltration of neutrophils and platelets into the myocardium. Atorvastatin was found to reduce both platelets and neutrophils in the previously ischemic region in B6/B6 and B6/KO chimeras, but not in KO/B6 chimera (Fig 5).

## Discussion

Many studies using *in vivo* animal models have consistently demonstrated that statins significantly reduce myocardial ischemia/reperfusion injury by activating eNOS [22]–[24]. However, the cell type(s) that statins act on remained unclear. By performing experiments in wild type (B6) and eNOS knockout (KO) mice, and chimeras of the two strains, we demonstrate here that bone marrow-derived cells are the primary mediators of myocardial reperfusion injury. These results are entirely consistent with our previous reports [19], [20]. Furthermore, the experiments performed in bone-marrow chimeras clearly demonstrate that the cardioprotective effect of atorvastatin is primarily due to its activation of eNOS in bone marrow-derived cells.

In wild type B6 mice, atorvastatin was found to significantly reduce myocardial infarct size and this salutary effect completely disappeared in eNOS KO mice; indicating that activation of eNOS mediates the effect of atorvastatin in reducing post-ischemic myocardial injury. In KO/B6 chimeras, which lack eNOS only in bone marrow-derived cells, the protective effect of atorvastatin was also abolished. In B6/KO and KO/KO chimeras, where endothelial cells are deficient of eNOS, peripheral arterial blood pressure and LVESP are significantly increased compared to B6/B6 and KO/B6 where endothelial eNOS is still intact. However, the infarct-sparing effect of atorvastatin persisted in B6/KO mice but not in KO/KO mice. The results are consistent with the conclusion that cardioprotection by atorvastatin is due to its effects on bone marrow-derived cells, not on the vasculature. Furthermore, immunostaining showed that atorvastatin markedly reduced the infiltration of platelets and neutrophils into the post-ischemic myocardium, indicating that atorvastatin protects the heart against reperfusion injury by inhibiting inflammatory responses through the activation of eNOS in bone marrow-derived cells.

It is well established that the inflammatory response elicited by myocardial I/R injury includes leukocyte adhesion to endothelial cells, followed by the transmigration of leukocytes into the interstitial space of the reperfused myocardium. Myocardial ischemia and reperfusion is also known to promote the emigration of neutrophils into myocardium upon restoration of blood flow to initiate a cascade of neutrophil-mediated injury. Reperfusion causes a dramatic increase in neutrophil adherence to the reperfused endothelium, which leads to capillary plugging and edema resulting in a reduction in coronary blood flow [19], [25]. The adhesion of neutrophils to endothelial cells is mediated by a well-defined sequence of interactions between cell adhesion molecules on both the endothelium and neutrophils. Recently, platelets were found to contribute to I/R injury by interacting with endothelial cells and enhancing neutrophil-induced I/R injury [26]–[28]. Platelets are among the first cells recruited within minutes after reperfusion and they colocalize with leukocytes in area of infarction [20]. In a recent study, platelet P-selectin (independent of endothelial P-selectin) was found to mediate neutrophil-induced myocardial injury [27], indicating a significant role for platelets in this process [29].

The potential role of statins to reduce acute myocardial I/R injury has not been clearly elucidated until recently. An increasing body of evidence has shown that statins appear to have pleiotropic effects beyond their ability to lower lipid levels [13], [30]–[32]. Although the signal transduction pathways have not been clearly defined, statins exert cardiovascular protective effects by improving endothelial function [30], [33]–[35], inhibiting inflammatory responses [6], [7], [36] and antagonizing thrombogenic tendencies [22], [37]. Atorvastatin and simvastatin improve endothelial function both by upregulating eNOS expression and by enhancing endothelial NO production [13], [31], [34]. The increase in ambient nitric oxide, in turn, inhibits the cell surface display of adhesion molecules, neutrophil accumulation and platelet aggregation [37]. Signal transduction pathways that might enhance nitric oxide production by eNOS in endothelial cells have been reported including the activation of PI3K and Akt [13], [23] and the inhibition of Rho GTPase [16]. Statins are now recognized to be potent anti-inflammatory drugs. A number of studies have reported very powerful anti-inflammatory actions of statins that are largely dependent on eNOS [13], [16], [31], [34]. The inducible isoform of NOS (iNOS) is also reported to protect against myocardial I/R injury [38] and to mediate the cardioprotective effects of atorvastatin downstream of eNOS [39]. However, iNOS in bone marrow derived leukocytes is reported to be deleterious during myocardial I/R injury [40]. One explanation for this apparent discrepancy is that NO produced by iNOS in different cell types mediates different biological functions. Further research is needed to elucidate the effects of atorvastatin on iNOS in bone marrow derived cells.

Recently, eNOS has been identified in human and mouse platelets [16], [17], and nitric oxide released from activated platelets inhibits platelet recruitment [41]. Atorvastatin significantly increased eNOS levels in platelets in a dose-dependent manner and also decreased platelet activation *in vivo*, which may contribute to atorvastatin-mediated protection against cerebral ischemia/reperfusion injury [16]. The role of platelet eNOS in limiting myocardial ischemia/reperfusion injury has yet to be explored. However, it will be particularly interesting to investigate given the current evidence suggesting that the putative antithrombotic and cardioprotective effects of statins are not exclusively due to modulation of the endothelial eNOS system. The current study for the first time clearly demonstrates that the infarct-sparing effect of atorvastatin is primarily due to its action on bone marrow derived cells, probably platelets. By using wild type B6 and eNOS KO mice, atorvastatin was found to exert cardioprotective effect via activation of eNOS. Atorvastatin was also found to reduce circulating neutrophils, which are widely considered to be the end-effectors of myocardial reperfusion injury. In order to differentiate between the specific roles of eNOS in endothelial vs. circulatory cells, eNOS tissue-specific knockout mice were created by bone marrow transplantation between B6 and eNOS KO mice. These chimeric mice demonstrated that endothelial eNOS plays a major role in regulating arterial blood pressure; however, eNOS in circulatory cells only played a minor role in this regard (Table 2). Although there were no significant differences in infarct size among vehicle-treated chimeras, atorvastatin significantly reduces infarct size only in those chimeras where functional eNOS is retained in blood borne cells (Figs 3&4). Further, inflammatory responses as reflected by the accumulation of platelets and neutrophils in the myocardium were alleviated only in those chimeras that retained functional eNOS in their blood borne cells (Fig 5). Thus our results do not support a role for cardiomyocyte eNOS in mediating the cardioprotective effects of atorvastatin. However, Bell et al. have reported that atorvastatin protects isolated perfused hearts by activating the PI3K/Akt pathway and phosphorylating eNOS [13]. As always, it should be noted that different animal models (ex vivo vs. in vivo) often give rise to conflicting results. Using an in vivo model, we here demonstrate that atorvastatin fails to protect chimeric mice that have eNOS in cardiomyocytes, but are deficient in bone marrow derived eNOS. Further, it should be noted that bone marrow derived immune cells resident in heart tissue have been reported to mediate pharmacological cardioprotection in isolated perfused hearts [42].

Statins are now recognized to be powerful anti-inflammatory agents that can exert cardiovascular protective effects by improving endothelial function, inhibiting inflammatory responses and antagonizing thrombogenic tendencies. Activation of eNOS is thought to be the mechanism primarily responsible for the anti-inflammatory properties of this class of drugs. Chronic treatment with statins exert their anti-inflammatory capacities as mentioned above by activating eNOS of myocytes, endothelial cells and bone marrow-derived cells, probably via the transcriptional activation [35], [43]. The efficacy of acute stain therapy shortly before reperfusion in reducing the size of myocardial infarction has been reported recently. The current studies further confirmed that acute administration of atorvastatin just prior to the onset of reperfusion significantly reduces myocardial reperfusion injury in an eNOS-dependent manner, probably through the post-transcriptional activation of eNOS. Interestingly, the cardioprotective effect of atorvastatin after chronic treatment wanes with time associated with an increase in PTEN levels. This waning protection can be recaptured by an acute high dose given immediately before ischemia and reperfusion [44]. So the cardioprotective mechanisms of statins are very likely different between the acute and chronic treatment. With acute use, the infarct-sparing effect of statin is primarily due to its action on bone marrow-derived cells through post-transcriptional activation of eNOS [45].

In summary, by testing the effect of a statin, atorvastatin, in wild type, eNOS knockout, and chimeric mice specifically lacking eNOS on bone marrow-derived cells, atorvastatin was found to play a critical role in down-regulating pro-inflammatory responses and mediate cardioprotection against reperfusion injury through the activation of eNOS. The infarct-sparing effect of atorvastatin is primarily due to its action on bone marrow-derived cells, probably platelets. These results may have potential clinical relevance. The significant reduction in infarct size achieved by adjunctive use of statins in conjunction with direct percutaneous coronary intervention (PCI) or thrombolytics has the potential to define a new standard in cardiac care.

## Supporting Information

Data S1 **Raw data of** **Figures 1** **,** **2** **,** **4** **and** **Table 2** **.**
(XLS)
